# A revisionist model for treatment-resistant and difficult-to-treat depression

**DOI:** 10.1177/00048674241240600

**Published:** 2024-03-27

**Authors:** Gordon Parker

**Affiliations:** Discipline of Psychiatry and Mental Health, University of New South Wales, Sydney, NSW, Australia

**Keywords:** Classification, treatment-resistant depression, difficult-to-treat depression

## Abstract

**Objective:**

The aim of this study is to consider limitations to the heuristics ‘treatment-resistant depression’ (TRD) and ‘difficult-to-treat’ depression (DTD) and to offer a revisionist model.

**Methods:**

A number of limitations to the two constructs are noted, particularly the risk of each positioning clinical depression as an entity and then applying a linear sequencing management model.

**Results:**

Arguing that clinical depression is heterogenous in nature (with categorical and ‘fuzzy set conditions), in cause and in response to treatment, allows an alternate model for addressing depressive conditions that are not readily responsive to treatment. A skeletal model for proceeding is offered for consideration and development.

**Conclusion:**

If such a model is accepted, then differing criteria for defining treatment resistance and treatment failure might be generated for differing depressive conditions, and condition-specific sequencing algorithms (embracing drug and non-drug strategies) developed for their management.

## Introduction

It is widely recognized that a high percentage of individuals with a clinical depressive episode do not respond to recommended therapeutic strategies. In a detailed review of treatment-resistant depression (TRD), McIntyre et al. (2023: 394) stated that ‘the majority of individuals with MDD [major depressive disorder] are inadequately responsive to first-line treatments. Moreover, a substantial proportion of them fail multiple antidepressant interventions, resulting in what is described as treatment-resistant depression (TRD)’. Similarly, in a paper considering ‘difficult-to-treat depression’ (DTD), Rush et al. (2022: 419) observed ‘Approximately one-third of individuals in a major depressive episode will not achieve sustained remission despite multiple, well-delivered treatments’. These quotes on prevalence also illustrate a key issue – that most current considerations of TRD and DTD position clinical depressive conditions as compassable by the Diagnostic and Statistical Manual of Mental Disorders (*DSM*) diagnostic construct of major depression, which is commonly conceptualized as an entity. Such a model risks sequencing treatment strategies that do not concede or consider differing depressive conditions. The objectives of this article are to review the TRD and TTD heuristics, argue that both are compromised by conceptualizing major depression as an entity and then offer a revisionist model – a task risking braggadocio in play.

## TRD conceptualized and defined

While the term TRD was introduced to capture and grade depressed patients who did not respond to antidepressant medication, it has mutated to commonly describe treatment failure in general and is quite variably defined. Brown et al. (2019) noted that a systematic review of published literature identified 155 definitions of TRD, while Wijeratne and Sachdev (2008) observed that there are no formal diagnostic criteria for TRD. Furthermore, McIntyre et al. (2023) drew attention to the lack of any consensus-derived definition of TRD possessing predictive utility.

The concept of TRD emerged in a seminal paper by Thase and Rush (1997). Their model focused only on antidepressant drug non-responders or those who could not tolerate the initial medication – and was therefore limited to those evidencing resistance to antidepressant drugs alone.

In borrowing an oncology staging model, Thase and Rush offered a five-stage model for addressing such ‘antidepressant resistance’. Stage I was defined as failure of at least one adequate trial of one major class of antidepressant. Stage II captured stage I resistance plus similarly defined failure to respond to an antidepressant of a differing class. Stages III, IV and V captured resistance across the previous stages and failure to respond to an adequate trial of a tricyclic antidepressant (TCA), a MAOI (monoamine oxidase inhibitor) or electroconvulsive therapy (ECT) respectively.

The Thase and Rush staging model has been largely sustained in guidelines for managing TRD, albeit with some definitional modifications. For example, McIntyre et al. (2023) noted that both the US Food and Drug Administration (or FDA) and the European Medicines Agency (or EMA) define TRD as failure to respond to two or more antidepressant drug regimens despite adequate dose and duration as well as adherence to treatment.

## TRD and its linkage to major depression

The common linkage of TRD to ‘major depression’ is a key model component inviting challenge and with the challenge weighted to the construct of major depression itself. That diagnosis emerged in the revisionist *DSM*-III manual, which adopted a unitary model of the depressive disorders – in essentially viewing clinical depression as a single condition varying in severity – and so leading to ‘major depression’ and a set of minor depressive conditions differentiated dimensionally by symptom number and duration parameters. Subsequently, we have observed many scientific papers commencing with statements along the lines of major depression being a disease, being severe, persistent and recurrent – and, in so doing, weighting biological causes and according major depression entity status.

Such entity status has been critiqued by many. For example, Kendler (2016) expressed concern about the reification of major depression as an ‘it’ while Goldberg (2011) critiqued its assumed entity status as failing to recognize the heterogeneity of clinical depression.

As considered later, major depression can indeed encompass disease-like constituent conditions (in that they have genetic and biological weightings) but it is not invariably a disease - with social and psychological factors predisposing to and/or precipitating multiple other depressive sub-types and syndromal patterns. Major depression is also not of necessity ‘major’ or ‘severe’. The *DSM*-III manual (American Psychiatric Association, 1980: p7) stated that operational criteria (for all its listed conditions) were set ‘at the lowest order of inference’. Criterion A required a ‘dysphoric mood’ for 2 weeks, including feeling ‘sad, blue . . . down in the dumps’ (p. 213), a symptom profile that would be generally achieved by those with a ‘normal’ depressed mood. Criterion *B* (which mandated four of eight listed items) status could be achieved by the individual having some level of (1) appetite change, (2) sleep disturbance, (3) drop in libido and (4) fatigue, and thus able to be met by those with mild or even normative mood states.

It is also not necessarily persistent (beyond the mandated 2 weeks in duration criterion). Wakefield and Schmitz (2013) reported that of a sample of individuals with an ‘uncomplicated’ episode of major depression (i.e. not being suicidal, psychotic, having psychomotor disturbance nor feelings of worthlessness), only 3.4% still met major depression criteria 12 months later – with that rate not significantly higher than the 1.7% rate of major depression in a comparison group of those having no history of depression. Major depression is also not necessarily recurrent. Wakefield and Schmitz (2013) reported that their sample of individuals experiencing major depression were no more likely to have another episode than those in the general population. Major depression is also a seemingly unreliable diagnosis. Formal trials (involving two or more raters) have quantified its low reliability – as overviewed by Kirk and Kutchins (1992). An exemplar study comparing *DSM*-III diagnoses made by lay interviewers and psychiatrists using a standardized interview schedule generated a low kappa coefficient of 0.25 (Anthony et al., 1985). In a subsequent *DSM*-5 filed study (Regier et al., 2013), the agreement in diagnosing major depression by two independent clinicians was also low (kappa being 0.25). Low reliability challenges the validity of an entity or construct, as defined and/or as measured.

As a reflection of its likely heterogeneity and impact on treatment options, Fawcett (2014: 414), the Chair of *DSM*-5’s Mood Disorders Work Group, observed that ‘right now, with major depression as our target . . . we are shooting in the dark’.

As staging criteria for TRD generally build on a foundation diagnosis of major depression, management recommendations are immediately compromised if major depression is instead heterogeneous – reflecting differing biological, psychological and social causal factors – and when differential responses to differing treatments would be anticipated. In addition, articles and guidelines detailing strategies for handling TRD generally evidence a sequence of linear treatment strategies (i.e. if drug strategy A fails for an individual with major depression, move to strategy B, and if that fails, move to strategy C, and so on). Such a linear sequential model further positions TRD as a (base) entity. The risk then is of TRD recommendations also risking ‘shooting in the dark’ status.

## Treatment failure or paradigm failure?

Weighting failure to respond to antidepressant drug therapy as a criterion for defining TRD is only logical if the condition being treated is theoretically responsive to an antidepressant drug or if the objective is to identify those depressive conditions responsive to and those resistant to medication.

Let us contemplate a few clinical scenarios. Vignette A: a woman who was physically and sexually abused in childhood, has been constantly humiliated by an abusive husband in recent years and lacking any financial resources is unable to leave the marriage. Vignette B: a patient with a distinctive borderline personality disorder who frequently enters brief dysfunctional relationships invariably ending with rejection by her partners. Vignette C: a man with a profoundly perfectionistic personality style and whose work superior publicly humiliates him for quite invalid reasons. Let us assume that all meet criteria for a current episode of major depression. Would we imagine that an antidepressant medication would be the first line of therapy for any of the three patients when causal factors seemingly weight psychological and social factors and thus invite non-drug strategies as the initial therapeutic modality? Accepting that a percentage of practitioners did prescribe two or more antidepressant drugs without benefit, most formal as well as informal criteria for TRD would be met – but would it not be more appropriate to judge that ‘paradigm failure’ was in operation as against ‘treatment failure’?

## Difficult-to-treat depression

Rush et al. (2019) introduced the heuristic term ‘difficult-to-treat’ depression or DTD (in a paper published in this journal) to capture those with major depression having a less-than-optimal outcome to treatment and argued for its advantages over TRD and seemingly a more useful descriptor or sobriquet for the three vignettes just provided. In a subsequent paper (Rush et al., 2022), the authors listed a lengthy set of challenges to the concept of TRD, including whether a lack of response or a lack of remission defines a failed trial, those instances whereby patients improve and then relapse, occasions when a medication cannot be tolerated and whether previously failed treatments should be included in defining TRD. They also noted that TRD definitions generally fail to include a non-pharmacological treatment (e.g. psychotherapy, psychosocial interventions). Importantly, they observed that ‘TRD has no practical, actionable clinical applications other than to suggest attempting another primarily pharmacological treatment trial with a different intervention or combination’ (p. 420). They argued that the heuristic DTD sought to overcome such limitations and that it had ‘been proposed to stimulate the timely identification and personalized management of patients for whom our current treatments . . . are unlikely to either initiate or sustain symptomatic remission’ (p. 420).

They provided a list of potential parameters to defining DTD and ‘to characterize subgroups’ (p. 422), including number, types and sequence of failed treatments, early life trauma, concurrent medications, degree of functional impairment, life history, symptom variability, adherence to treatment, symptom features, course of depression and both general medical and psychiatric comorbid conditions. They did not, however, consider the limitations imposed by a foundation diagnosis of major depression.

## Acquired entity status of TRD and other issues in play

As noted earlier, guidelines for managing TRD generally lay out a sequential or linear approach principally involving physical therapies (e.g. drugs and ECT), which commonly also now evaluate more recently introduced physical treatments such as ketamine and repetitive transcranial magnetic stimulation. There are two issues in play as a consequence. First, just as ‘major depression’ has acquired the entity status, any linear therapeutic regime with a base diagnosis of major depression effectively risks also positioning TRD as an entity. Adopting the Thase and Rush (1997) oncology reference, such an approach is akin to positioning a single ‘major cancer’ condition, then applying a linear sequence of therapies (say surgery first, then chemotherapy and then radiotherapy – or any other sequence) and then independent of the logic of the sequence in relation to any specific cancer, imposing interval ‘treatment-resistant cancer’ (or TRC) stages. The second concern is one of biological determinism being in play. Non-physical therapies are either not listed in the management of TRD or positioned as augmenting and complementing strategies and without any specifics detailing any nuanced contribution they might make.

I suggest again that the rate-limiting step to developing any model for managing TRD (and of some relevance to DTD-defined depression) is viewing major depression as an entity. Such a concern has clearly been noted by key authors in relation to TRD. For example, Wijeratne and Sachdev (2008: 753) critiqued ‘conceptualizing depression as a unitary disorder’ and noted the rarity of authors conceding heterogeneity in ‘depression’ in those meeting TRD criteria. There are, however, exceptions. Rush et al. (2022: 426) observed that ‘DTD and TRD are heterogenous in clinical presentation’ and noted the ‘current lack of taxonomy’ for DTD. Furthermore, they argued for a DTD taxonomy that ‘could facilitate the identification of more homogeneous subgroups’ (p. 421). McIntyre et al. (2023: 395) noted cogently in their recent major review of TRD that ‘the pathway towards more targeted treatments in psychiatry requires a more precise delineation of the phenotype being evaluated’.

If major depression (as for ‘clinical depression’) is not an entity and is, in essence, a domain diagnosis effectively homogenizing intrinsically heterogeneous constituent conditions, then there is a strong argument for respecting depressive phenotypes as a primary component in any TRD or management model.

As noted, Thase and Rush (1997) adopted an oncology model in their staging model for TRD. But any equivalent model in oncology would first define the type of cancer, and then, if sequential treatment models were in play, it would be expected that they would differ for differing cancers.

## An alternate model

An alternative strategy (both for the management of depressive disorders in general and for those evidencing resistance or being difficult to treat) is to allow the idea that clinical depression comprises several depressive sub-types and a number of clinically meaningful (albeit ‘fuzzy set’) depressive syndromes and that each are likely to show differing response gradients to differing therapeutic modalities. Respecting that view would allow primary and subsequent therapeutic strategies to be identified for each ‘phenotype’ and also potentially allow condition-specific treatment-resistant stages and difficult-to-treat scenarios to be identified.

This model predictably invites the question as to whether there are clinically meaningful depressive phenotypes. The 2020 RANZCP guidelines (Malhi et al., 2021) provided one (ACE) model, partitioning mood disorders into ‘activity’, ‘cognition’ and ‘emotion’ (ACE-based) states, and provided one model for identifying phenotypes. I offer the following set, limited to unipolar conditions and ignoring clinical depressive episodes secondary to physical conditions or to drug and alcohol problems.

## Utility of the ‘binary’ model?

Prior to *DSM*-III’s introduction of major depression in 1980, a binary model had long been evident with contrasting ‘endogenous/melancholic/vital’ and ‘neurotic/reactive’ depressive types. There is support for melancholic depression being a categorical type, as shortly considered, but an argument for the listed second ‘type’ is difficult to mount, and thus, ‘it’ is better viewed as a category capturing multiple heterogeneous and residual non-melancholic depressive conditions.

The case for melancholia being a categorical type rests on four long-standing ascriptions (see Parker et al., 1996): that it has a distinctive pattern of symptoms and signs, the greater relevance of primary genetic and other biological determinants, concomitant evidence of perturbed biological functioning and it showing a selective preferential response to physical treatments such as antidepressant medications and ECT. If it is a valid entity, then it should conform to most if not all of those ascriptions. A review (Parker, 2021) identified several studies supporting a strong genetic contribution. Another review (Spoelma et al., 2023) identified a very large number of biological markers over-represented in those with melancholic depression. Assuming for the moment that it is an entity, then lack of any acceptance of such status is likely a reflection of difficulties in defining and measuring ‘melancholia’. Rush and Weissenburger (1994) examined nine diagnostic candidates (including *DSM*-III and *DSM*-III-R criteria) and found little agreement between their criteria. The only common feature in all systems was psychomotor retardation.

Since the Rush and Weissenburger review, several new measures of melancholia have been developed including the Sydney Melancholia Prototypic Index, with clinician-rated (SMPI-CR) and self-report (SMPI-SR) versions (Parker et al., 2013) and with the former version being quantified as having 98% prediction accuracy in identifying melancholic depression (Parker and Spoelma, 2021). The importance of defining and measuring melancholia with precision lies in evaluating long-term ascriptions for treatment for its forerunner ‘endogenous depression’. Of key relevance in this instance is that multiple studies demonstrated that those with endogenous depression responded better to antidepressant drugs and to ECT than those with non-endogenous or non-melancholic depression (see Parker and Hadzi-Pavlovic, 1996). Furthermore, endogenous depression showed a differential response to differing classes of antidepressants, with Perry (1996) quantifying a three-times-higher response rate to broad-action TCAs than to selective serotonin reuptake inhibitors. Thus, there is support for viewing melancholia as a categorical type.

The status of psychotic or delusional depression remains unclear with authors variably positioning it as a ‘more severe’ expression of melancholia or as a distinct entity. Either model would still allow it to be positioned as a categorical type (whether as ‘psychotic depression’ or as ‘melancholia with psychotic features’). A Cochrane review (Smith, 2017) determined that an antidepressant plus an antipsychotic was more effective than either drug type alone, while an earlier analysis (Spiker et al., 1985) concluded that its response to ECT was comparable to the antidepressant/antipsychotic drug combination therapy. Thus, independent of its status, it would appear worthy of separate consideration in developing any revisionist model for managing TRD or depression.

Turning to the residual non-melancholic depressive conditions (historically termed ‘neurotic’ or ‘reactive’ depressions), as noted, they appear to lack categorical status and are more ‘fuzzy set’ syndromes rather than sub-types. Candidate conditions include stress-related depressive conditions (with acute, chronic and acute-on-chronic expressions) and conditions reflecting a predisposing personality style (e.g. anxious worrying, avoidant, perfectionistic, interpersonal sensitivity to rejection and low self-esteem). Thus, primary social and psychological factors appear to be the principal determinants of the non-melancholic conditions. For each of the syndromal presentations, it might be expected that there would be preferential initial treatment options and candidate subsequent options awaiting specification and sequencing.

Such an approach differs not only in its attempt to offer the potential saliency of a broad set of candidate treatments (not limited to physical treatments) but also in arguing for treatments weighted to the ‘driver’ of the particular condition. In psychiatry, a diathesis stress model is dominant. For those who have a melancholic or psychotic depressive condition, we assume that a physical treatment should logically address the perturbed biological pathways that have caused the individual to becoming depressed. For those with a non-melancholic depressive condition, the predisposing diathesis factor is commonly personality based, which argues for the therapy addressing the driver as much if not more so than simply addressing the precipitating stressor. For those who have an acute or chronic stress-induced non-melancholic depression, a personality driver may not be present, but there may still be diathesis factors (e.g. young age, low resilience, a lack of mature coping repertoires).

**Figure 1.**
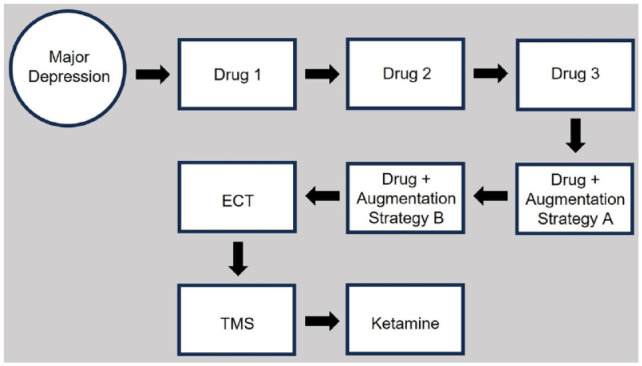
Standard model for conceptualizing TRD and its management, illustrating a base diagnosis of major depression capturing clinical depression as an entity and then providing a linear set of sequential treatment strategies. TRD status is then imposed at some sequence stage.

In Figure 1, an exemplar of the current TRD model is portrayed. It captures representative strategies of first trialling several antidepressant drugs and subsequent trialling of electroconvulsive therapy (ECT), transcranial magnetic stimulation (TMS) and ketamine (albeit with the ordering of the last three varying across multiple published exemplar models). The constituent treatments and their ordering are not the salient issues in play here. The figure simply seeks to demonstrate two key components – depression positioned as an entity (here ‘major depression’) and treatment options being listed according to a linear sequencing model.

**Figure 2.**
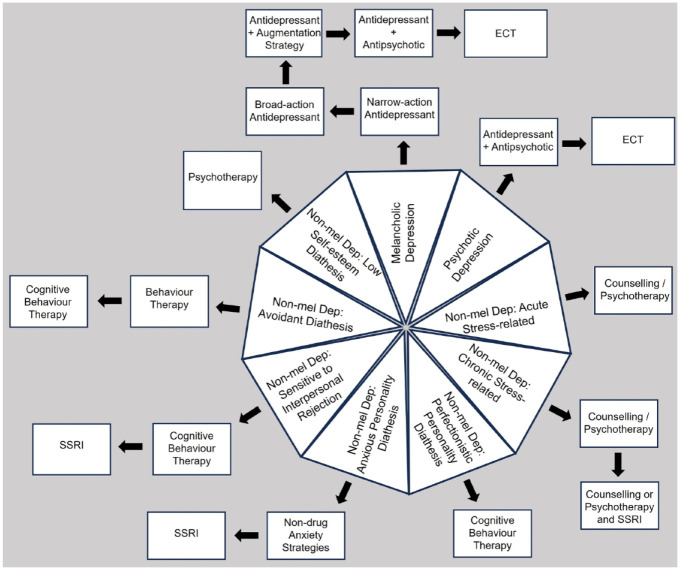
Proposed model for conceptualizing TRD and its management illustrating clinical depression as comprising multiple constituent disorders and with each disorder having differing first option and subsequent management options (albeit limited here), and with some conditions weighting drug therapy initially while others weight non-drug strategies. TRD and depression status may then be imposed for each treatment sequence for each condition. Non-mel Dep = non-melancholic depression.

Figure 2 captures a revisionist model for conceptualizing TRD and also DTD depression. The base condition is ‘clinical depression’ and is captured by a pie chart with constituent conditions including both categorical expressions (i.e. melancholic and psychotic depression) and a representative set of non-melancholic conditions. For each condition, there are minimalistic sets of first, second and occasionally additional treatment options. None, alone, in number, in their initial prioritizing stage nor their sequencing, are offered as definitive. Instead, the salience of the model lies in simply suggesting that quite differing treatments are prioritized for the differing constituent conditions, some initially favouring a medication, and others favouring a psychotherapy or even counselling. Such a model clearly weights primary aetiological drivers but could well be extended by including secondary drivers. For example, at the clinical level, a patient may have a melancholic depression, but the consequences of it may result in a marital breakdown and loss of job, with those social factors compromising primary management of the melancholia.

## Quo vadis

If such an alternate model were to be accepted, then there would need to be some level of agreement on the candidate conditions to be listed and on recommended first, second and subsequent options for their management (ideally evidence-based ones). For each categorical or fuzzy set ‘condition,’ TRD stages could be imposed as well as nuances identified for management application.

## Conclusion

Applying a phenotype-weighted model differs from the standard model in considering TRD. First, it rejects major depression as an acceptable base diagnosis and argues for a need to respect depressive heterogeneity just as no model for managing TRC would list treatment strategies without first listing the type of cancer being considered. Second, TRD models focus on addressing the ‘depression’ (i.e. the outcome variable), whereas management of many depressive syndromes (and the model weighted here) benefits from also addressing the diathesis driver (e.g. certain personality styles) or neutralizing the precipitating factors.

Both the TRD and DTD heuristics are intrinsically attractive to clinicians and deserve field status. The aim of this paper is to argue for their application and further development within a nosological model that views clinical depression as heterogenous in phenotype, cause and likely responsivity to differing treatment modalities.
